# The role of toll-like receptor 4 in tumor microenvironment

**DOI:** 10.18632/oncotarget.19105

**Published:** 2017-07-08

**Authors:** Jing Li, Fan Yang, Feng Wei, Xiubao Ren

**Affiliations:** ^1^ Department of Immunology, Tianjin Medical University Cancer Institute and Hospital, Tianjin, China; ^2^ Department of Biotherapy, Tianjin Medical University Cancer Institute and Hospital, Tianjin, China; ^3^ National Clinical Research Center for Cancer, Tianjin, China; ^4^ Key Laboratory of Cancer Prevention and Therapy, Tianjin, China; ^5^ Tianjin’s Clinical Research Center for Cancer, Tianjin, China; ^6^ Key Laboratory of Cancer Immunology and Biotherapy, Tianjin, China

**Keywords:** TLR4, immune cells, tumor cells, tumor microenvironment

## Abstract

Tumors are closely related to chronic inflammation, during which there are various changes in inflammatory sites, such as immune cells infiltration, pro-inflammation cytokines production, and interaction between immune cells and tissue cells. Besides, substances, released from both tissue cells attacked by exogenous etiologies, also act on local cells. These changes induce a dynamic and complex microenvironment favorable for tumor growth, invasion, and metastasis. The toll-like receptor 4 (TLR4) is the first identified member of the toll-like receptor family that can recognize pathogen-associated molecular patterns (PAMPs) and damage-associated molecular pattern (DAMPs). TLR4 expresses not only on immune cells but also on tumor cells. Accumulating evidences demonstrated that the activation of TLR4 in tumor microenvironment can not only boost the anti-tumor immunity but also give rise to immune surveillance and tumor progression. This review will summarize the expression and function of TLR4 on dendritic cells (DCs), tumor-associated macrophages (TAMs), T cells, myeloid-derived suppressor cells (MDSCs), tumor cells as well as stromal cells in tumor microenvironment. Validation of the multiple role of TLR4 in tumors could primarily pave the road for the development of anti-tumor immunotherapy.

## INTRODUCTION

TLR is a type I transmembrance protein, which contains an extracellular domain and intracellular domain. At present, 11 mammalian TLRs have been identified. TLR4 is one of the most studied TLRs [1]. TLR4 was identified in Drosophila as a factor related to dorso-ventral body patterning, and latter found in human body by Janeway and Medzhytov in 1997 [1]. Once being activated, TLR4 mainly initiates two different downstream pathways: MyD88-dependent and MyD88-independent signaling pathway. MyD88 activates transcription factors, containing nuclear factor-kappa B (NF-κB) and activator protein-1 (AP-1), through IκB kinase (IKK) and mitogen-activated protein kinase (MAPK) pathway, which are favorable for transcription of inflammation factors. The other way is mediated by Toll/IL-1R domain-containing adapter-inducing interferon-β (TRIF) and toll receptor-associated molecule (TRAM) [2], which lead to expression of IFN-α and IFN-β, playing a role in resistance to virus. The TLR4 signaling pathway can be stimulated by varies ligands of TLR4 such as PAMPs and DAMPs *in vivo* [3, 4]. Among which, DAMPs rather than PAMPs play a main role in activating TLR4 in tumor microenvironment. TLR4 and MyD88 have been demonstrated over expression in breast cancer [5], suggesting the possible role of TLR4 signaling pathway in tumor microenvironment. TLR4 is expressed not only on tumor cells but also on stromal cells and immune cells that play vital role in antitumor in tumor microenvironment. Many TLR4 ligands have been identified in tumor tissue in recent years. Nevertheless, a growing body of evidences have pointed out the existing of immune inhibitory cells, such as TAMs, and MDSCs, which are considered as the major factors for the suppression of immune response in tumor microenvironment. Of note, macrophages in tumor microenvironment were found to show pro-tumor or anti-tumor functions in various studies [6–9]. Accumulating evidences demonstrated that the activation of TLR4 in tumor microenvironment can boost the anti-tumor immunity [10–12] including dendritic cells (DCs) maturation, and antigen presentation. However, TLR4 activation on tumor or on stromal cells promotes immune surveillance and tumor progression [13]. NF-κB is active in macrophages during the stimulation of inflammatory cytokines and partly contributes to tumor progression. A positive correlation between abundant TAMs and poor prognosis has been demonstrated by a great amount of clinical studies concerning prostate cancer [14], gliomas [15] and non-small lung carcinoma [16]. In Yusuf’s study, TLR4 deficient mice are more susceptible to cutaneous 7,12-dimethylbenz induced carcinogenesis, indicating a role of TLR4 in preventing tumorigenesis [17]. In another relevant study, effective chemopreventive agent was used before 7,12-dimethylbenz, and IFN-γ and IL-12 levels were increased in TLR4 competent mice compared to TLR4 deficient mice, suggesting that TLR4 is an important mediator of chemoprevention in 7,12-dimethylbenz skin tumorigenesis [18]. Furthermore, activation of TLR4 on DC by adjuvants including BCG cell-wall skeleton (BCG-CWS), M. tuberculosis protein Rv0652 [19], Heat shock protein X (HspX), Hyaluronan [20], and Angelan [21, 22], leads to enhanced maturation and migration of DCs, macrophage activation, naïve T cell proliferation as well as Th1 immune response, which all play a positive role in antitumor immune therapy. Additionally, TLR4 signaling has also been shown to partially reconstruct destroyed vasculature at the site of damage by recruiting the provascular progenitors from bone marrow (BM) and spleen to inflamed tissue [23]. high-mobility group box-1 protein(HMGB1), ligand of TLR4 has been reported correlation to integrin-dependent homing of endothelial progenitor cells [24]. Interestingly, the similar effect of TLR4 on angiogenesis is also discovered in tumor microenvironment. Therefore, this review focused on the expression and function of TLR4 on DCs, TAMs, T cells, MDSCs, tumor cells and stromal cells in tumor microenvironment.

### The effect of TLR4 on DCs activation and maturation

**Figure 1:**
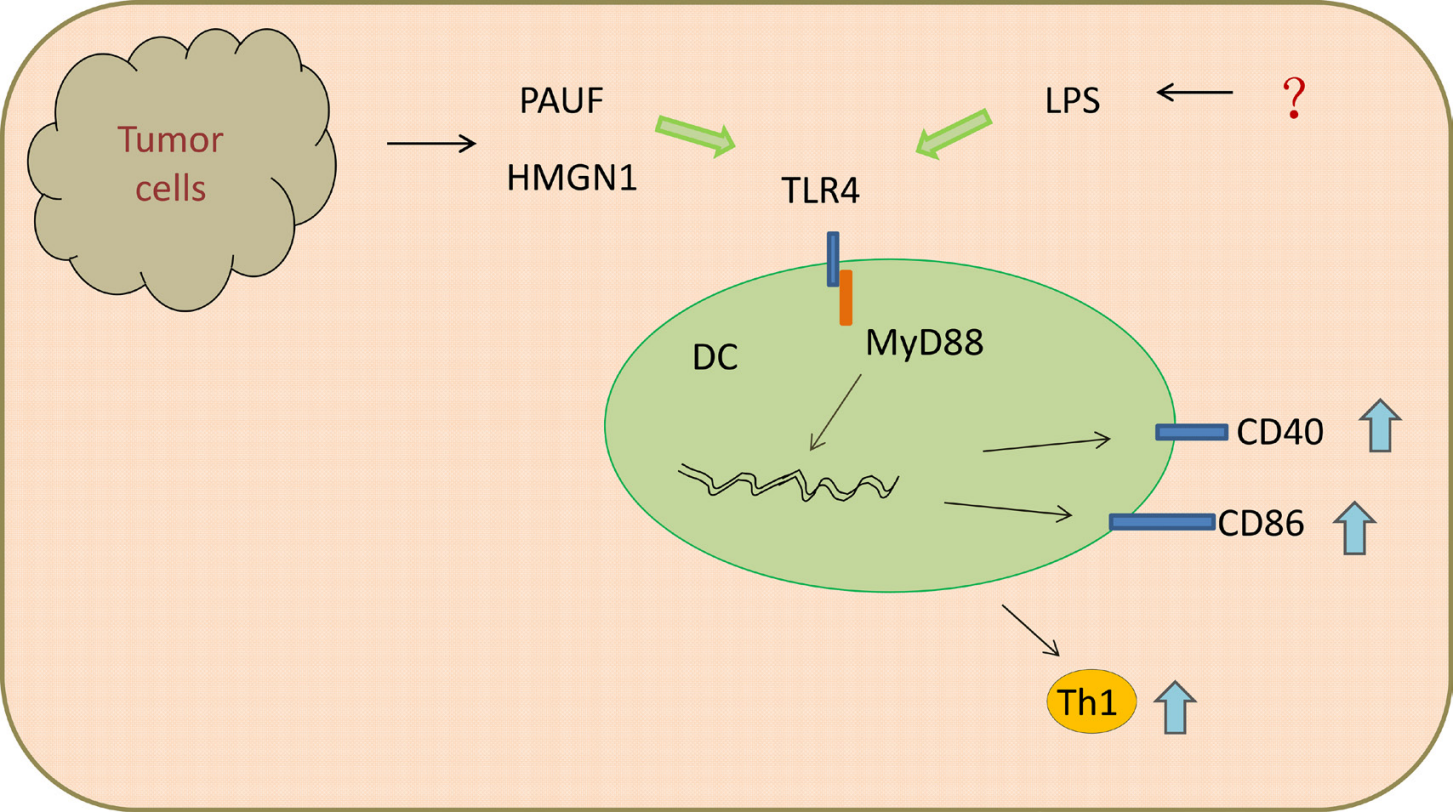
Ligands of TLR4 promoted DCs maturation and Th1 anti-tumor immunity through MyD88-dependent pathway

DCs act as the most powerful antigen presenting cells (APCs) and critical mediators of adaptive immune responses. The two main populations of DCs recognized in mouse and human tissues are myeloid DCs (mDCs) and plasmacytoid DCs (pDCs). Among them, the expression of TLR4 is restricted to mDCs. Following LPS stimulation, DCs separated from pigs presented an enhanced TLR4/MyD88-dependent gene expression, CD40 and CD86 on the cell surface, as well as chemokine and pro-inflammatory cytokine expressions [25] (Figure 1). Mature DCs are able to induce T helper 1 (TH1) -type immune responses, and are also viewed as potent initiators of tumor associated antigen (TAA)-specific immunity. Interestingly, Davis *et al.* [26] found that intratumoral treatment of LPS, acting as TLR4 agonist, combined with granulocyte-macrophage colony-stimulating factor (GM-CSF) gene modified tumor vaccine (GVAX), enhanced maturation of APC in tumor microenvironment and induced efficient anti-tumor response. Small difference was found in mice with MyD88^−/−^ as well as MyD88^−/−^TRIF^−/−^ genotypes between GVAX and PBS treated group, suggesting this process was in a TLR4-dependent pathway. Additionally, an ocean of adjuvants, such as pancreatic adenocarcinoma up-regulated factor (PAUF) [27], Polysaccharides [28], high-mobility group nucleosome-binding protein 1 (HMGN1) [29], angelan [22], can induce activation and maturation of DCs through TLR4 (Figure 1).

However, growing evidences demonstrated that functional mature DCs are rare in human cancers. This may result from various factors, such as lack of DC recruitment, differentiation, activation, maturation, or survival. It was note that both differentiation and maturation of mDCs can be suppressed by imbalance of cytokines in the tumor microenvironment. Furthermore, Zhong *et al.* [30]also found that mDCs can be polarized into functional regulatory DCs (regDCs) in the lung cancer microenvironment *in vitro* and *in vivo*. There was also a suppressed proliferation of pre-activated T cells with the development of regDC, which were confirmed with a potential pro-tumorigenic activity in tumor-bearing mice [30]. Furthermore, the progression of lung cancer is relevant to fast and significant accumulation of immunosuppressive regDC in both lymphoid tissues and tumor microenvironment [30]. The transformation from conventional DC (conDC) to regDC has been blocked by the use of noncytotoxic doses of paclitaxel through the small Rho GTPase signaling in a TLR4 independent way [30]. In consideration its crucial role in DC activation [31], maturation [32], differentiation [32, 33] as well as migration [34], TLR4 may be a underlying member in transformation between conDCand regDC, though there was no evidence on relationship of regDC and TLR4 at present.

### TLR4-induced inflammation in tumor leads to the infiltration of M2 macrophages

Macrophages in tumor microenvironment are often divided into two classically activated macrophages (or M1) and alternatively activated macrophages (or M2). The M1 phenotype is polarized by Th1 cytokines such as IFN-γ and characterized by high capacity, high levels of inflammatory cytokines secretion, so it enhanced ability to kill intracellular pathogens and tumor cells. In contrast, M2 macrophages are induced by Th2 cytokines such as IL-4 and IL-13, and marked by decreased production of IL-12 and increased production of IL-10 [35]. TAMs, important constituents of tumor microenvironment, have been regarded as M2 phenotype, playing a key role in promoting tumorigenesis and progression. For decades, accumulating evidences has revealed a positive correlation between advanced number and/or density of macrophage in tumors and poor prognosis of sufferers [35, 36].

**Figure 2:**
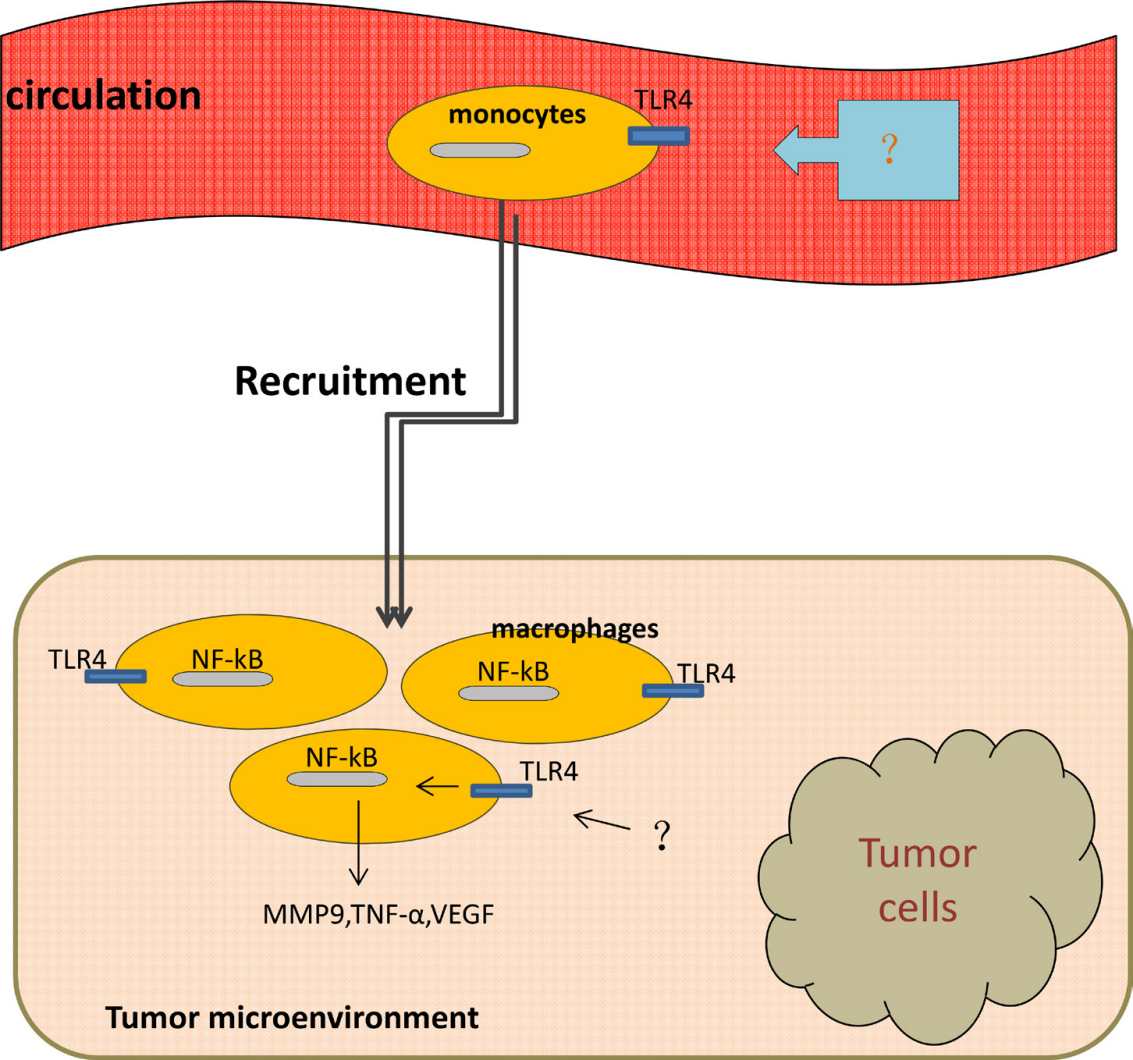
Macrophages move from circulation to tumor microenvironment with the activation of TLR4 on them Once been activated, TLR4/NF-κB signaling pathway induces production of MMP-9, TNF-α, VEGF by tumor-associated macrophages.

Lee *et al.* [37] have found a potential role for TLR4 signaling in inflammation and regulating the permeability of the lung tissue by comparing the levels of proteins in the bronchoalveolar lavage (BAL) fluid. Simultaneously, a 1.69-fold amplification of the number of macrophages exists in the BAL fluid stemmed from wild-type mice bearing melanoma when compared with that from tumor-bearing TLR4-deficient mice, the result suggested that TLR4 may play a role in macrophage migration [37]. Studies have also shown that activation of NF-κB in TAMs is essential for the production of pro-inflammatory cytokines and angiogenetic factors, such as metal matrix proteinase 9 (MMP-9), TNF-α, and vascular endothelial growth factor (VEGF) [37–39] (Figure 2).

In a recently study, a larger number of TAMs has been observed in mice exposed with sleep fragmentation (a hallmark of sleep apnea and associated with increased tumor incidence and mortality) compared with that in mice exposed with sufficient sleep [40], at the same time, TAMs secreted from the tumor of mice with sleep fragmentation had a higher level of TLR4 expression than that from the tumor of the latter, which indicates that TLR4 signaling in macrophages may play a role in recruiting macrophages from systemic circulation to the tumor microenvironment [40] (Figure 2). Significant reductions in TAMs count appeared in TLR4^-^/^-^ mice, indicating the potential role of TLR4 in macrophage recruitment [40]. A retrospective study on 81 patients diagnosed with diffuse large B-cell lymphoma (DLBCL) has shown a significant correlation between TAMs and TLR4 by immunohistochemistry experiment, this study implied that TLR4-induced inflammatory may be responsible for the TAMs collection in tumor microenvironment [41]. Above all, TLR4 expressed on TAMs is one component of mechanisms responsible for the recruitment of macrophages into tumor microenvironment.

### The relation between TLR4 and T cells in tumor microenvironment

CD4+ T cells can be mainly differentiated into Th1, Th2, T helper 17 (Th17), regulatory T (T_reg_) as well as T follicular helper cells, all of which play a dynamic role in immune responses to not only infectious diseases but also cancer. The Th1 response can be enhanced by a variety of TLR4 agonists, such as glucopyranosyl lipid A-stable emulsion (GLA-SE) and LPS. Studies have found that synthetic GLA can definitely upregulate the CD4+ T cell response with increased production of IFN-γ and TNF [42, 43]. Consistently, recently studies verified that adjuvant GLA-SE led to the existence of CD4+ CTLs, newly found members with the function of killing autologous B cells presenting MHC II complex [44], suggesting the momentous character of TLR4 in activating CD4+CTLs. This newly defined function of CD4+ T cells relies on CD40L engagement of CD40 in target cells rather than previously discovered certain CTL mechanism [44]. CD4 CTL expressed some markers related to cytotoxic functions of CD8 CTL, such as natural killer group 2 (NKG2A) and NKG2D [45, 46]. Considering the role of CD4 CTLs in controlling HIV, malaria, and other infections [47, 48], it is intriguing to speculate about the activity of CD4 CTLs in tumor environment after TLR4 agonist adjuvant. Nevertheless, a review has also revealed that CD4 CTL affect both protective and pathogenic immunity, so the function of CD4 CTL on tumor cells in tumor microenvironment should be ulteriorly researched by more studies [49].

Moreover, a study comparing the phenotype of human CD4^+^CD25^+^CD127^-^ T_reg_ and CD4^+^CD25^-^CD127^-^ conventional T cells (T_con_) showed that levels of TLR4 expressed on T_reg_ and T_con_ are similar, different with that of the receptor for advanced glycation end products (RAGE), which is higher on T_reg_ than on T_con_ [50]. The HMGB1, ligand of both TLR4 and RAGE, has been found to dampen the proliferation of CD4^+^CD25^-^T_con_ through activating TLR4 [50]. On the other hand, HMGB1 dramatically enhanced the IL-10 secretion of T_reg_ [50]. Additionally, the enhanced release of IL-10 was inhibited when anti-TLR4 antibody was added before or during HMGB1 stimulation [50], confirming that TLR4 is responsible for the elevated production of IL-10. IL-10 is a well known immunosuppressive cytokine in tumor microenvironment, and its increased levels are correlated with poorer prognosis of cancer patients [51, 52]. Furthermore, Liu *et al.* [53] has also revealed that the previous enhanced IL-10 production can further lead to the suppression of CD8+ T cell-dependent antitumor immunity in tumor microenvironment. Studies have shown that the number of tumor-infiltrating T cells was positive correlated with secretion of HMGB1 by cancer cells [54], specialists speculated that the underlying mechanism may be related to activation of TLR4 by HMGB1 in tumor microenvironment. In aggregate, activation of TLR4 on T cells by ligands in tumor microenvironment might have both anticancer and pro-tumor consequences through multiple mechanisms.

### TLR4 accounts for the accumulation and function of MDSCs in tumor microenvironment

**Figure 3:**
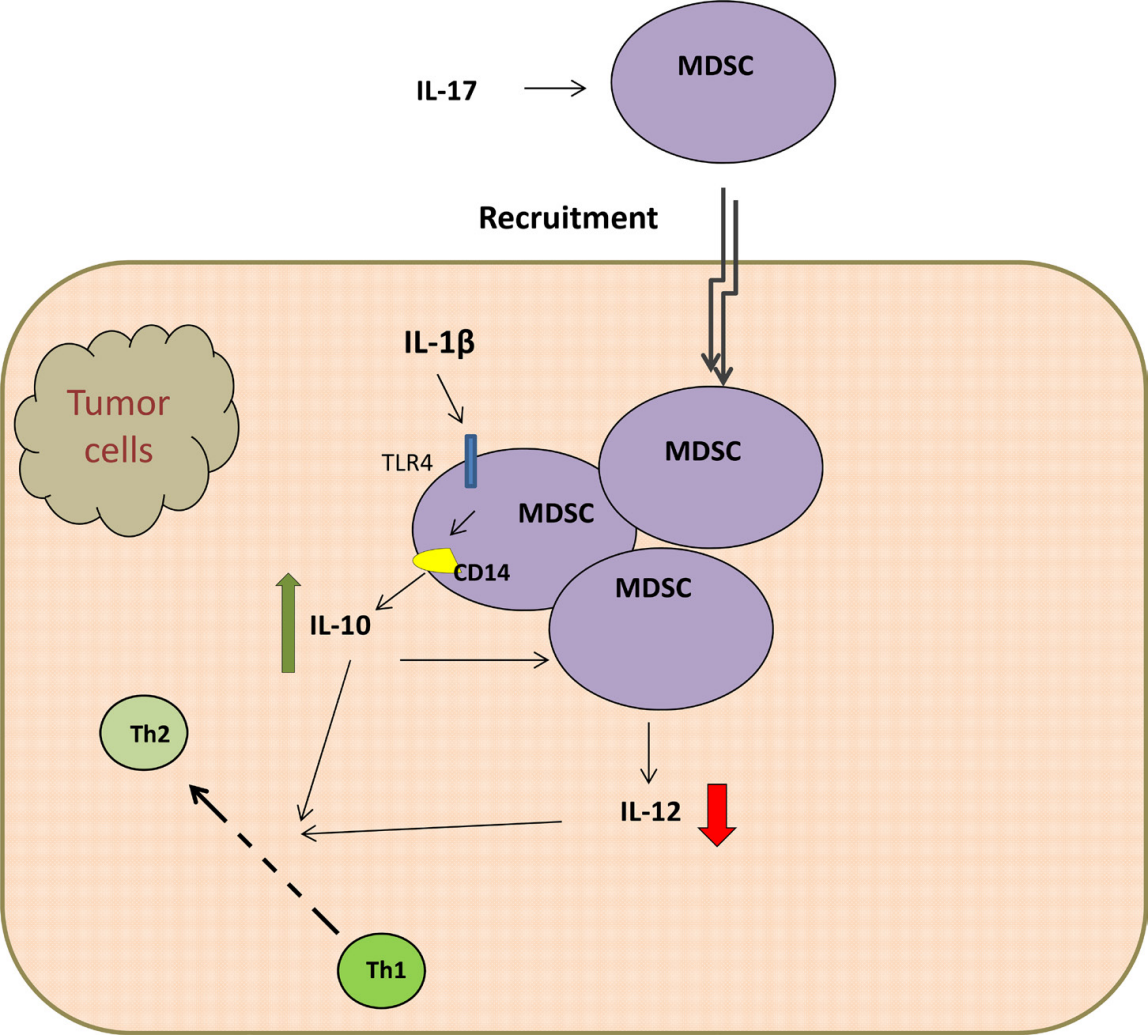
TLR4 / CD14 protein pathway elevated production of IL-10 by MDSCs and down-regulated IL-12 production, which both convert tumor immunity from a tumor-rejecting type 1 response to a tumor-promoting type 2 response

MDSCs, a heterogeneous population of immature myeloid cells, are known for its immune-suppressive function during chronic inflammation and tumor development. MDSCs are defined as Gr-1^+^CD11b^+^ cells comprising pathologically activated CD11b^+^Ly6C^low^Ly6G^+^ immature granulocytes and CD11b^+^Ly6C^high^Ly6G^-^ monocytes [55, 56]. MDSCs can be induced by pro-inflammatory cytokines such as IFN-γ, interleukins, GM-CSF, and TNF. However, the accumulation and function of MDSCs in the tumor microenvironment has not been well clarified. Chronic inflammation has been demonstrated to boost the aggregation of MDSCs. One recent study has testified that IL-17 promotes MDSCs recruitment in lung cancer induced by K-ras mutation [57] (Figure 3). Studies have also manifested that MDSCs played a role in inhibiting anti-tumor immunity by abrogating the activation of CD4+ and CD8+ T cells, the activity of macrophages and NK cells, and the maturation of DCs. Bunt *et al.* [58] reported that MDSCs can be activated by IL-1β-induced inflammation through the TLR4 / CD14 protein pathway, which enhanced the interaction between MDSCs and macrophages (Figure 3). This is the first study that has reported the expression of TLR4 in MDSCs. In addition, the suppressive activity of MDSCs needs to be induced by activated T cells and tumor stroma cells, such as IFN-γ, TGF-β, IL-13, IL-4, and certain ligands for TLRs [59]. The elevated production of IL-10 by IL-1β-induced MDSCs and down-regulated production of IL-12 converted tumor immunity from a tumor-rejecting type 1 response to a tumor-promoting type 2 response [59] (Figure 3), which promotes tumor growth. Srivastava *et al.* showed that a significant increase of MDSCs resulted from the injection of Lewis lung carcinoma (LLC) cells into C57BL/6 mice [60]. Of note, there is a inhibition of tumor volume, tumor weight as well as metastases and a reduction of Gr1-expressing cells in the tumor, blood, spleen and bone marrow after the application of anti-Gr1 or anti-Ly6G [60]. Moreover, the expression of TLR4 protein as well as the number of CD11b^+^Gr1^+^ cells in lung tissue are higher in urethane-treated mice than that in lungs of control group [61], which indicates that TLR4 may involve in the accumulation of MDSCs. Meanwhile, monophosphoryl lipid A, derived from LPS and also defined as a TLR4 agonist, led to a significantly accumulation of MDSC *in vitro* and *in vivo* [62].

Gr-1^+^CD11b^+^F4/80^+^ cells have been defined as another type of MDSCs in tumor-bearing mice, and have also been found with the capacity of inducing apoptosis of activated T cells [63]. Naive Gr-1^+^CD11b^+^F4/80^+^ bone marrow derived monocytes (BMDMs) in palpable tumor could not inhibit T cell activation without the stimulation of molecules from necrotic tumor cells (NTC-Ms) [64]. However, further study showed that interferon regulatory factor 3 (IRF-3), but not NF-κB, was activated in BMDMs in response to NTC-Ms, indicating that TRIF pathway was involved. The ability of BMDMs, stimulated by NTC-Ms, to induce apoptosis of activated T cells was suppressed by an inhibitor of TRIF pathway [65], suggesting that TLR4 ligands in NTC-Ms aroused the apoptosis-inducing capability of Gr-1+CD11b+F4/80+ BMDMs, which was further confirmed by the use of sTLR4 expression vector in palpable tumor [64]. PAUF, a soluble protein involved in pancreatic tumorigenesis and metastasis, interacted with TLR4 on the surface of MDSCs and enhanced their immunosuppressive effects on activated T cells in tumor microenvironment [66]. Furthermore, wilde et al. have found that repetitive injections of LPS induced a cluster of MDSC-like cells, which mediate suppression of T cell response on vitro [67]. In one word, TLR4 may be in charge of MDSCs accumulation and enhance its capacity of inducing-apoptosis of T cells.

### TLR4 promotes angiogenesis in tumor microenvironment

Angiogenesis, the formation of new blood vessels mediated by endothelial cells (ECs) and endothelial progenitor cells (EPCs), both of which can migrate to tumor cells, after that, proliferate and act as active factors in tumor microenvironment. Elevated TLR4 messenger RNA expression of blood vessels was found in antiphospholipid syndrome (APS) associated with increased rates of cardiovascular morbidity and mortality [68], suggesting a role of TLR4 in angiogenesis regulation. Lin *et al.* [69] firstly demonstrated that TLR4 may involve in angiogenesis induced by HMGB1. When combined with HMGB1, TLR4 expressed on neutrophils or macrophages activates IkB kinase (IKK) -β and IKK-α, and leads to the activation of NF-κB, resulting in the release of VEGF [70], a crucial mediator in pathological angiogenesis. In addition, Zhu *et al.* [71]*.* compared the effect of all-thiol form (at-HMGB1) and disulfide-HMGB1 (ds-HMGB1), two different redox states of HMGB1, on angiogenesis of colorectal carcinoma, and showed that at-HMGB1 stimulates ECs migration through interacting with RAGE, and ds-HMGB1 induced VEGF-A secretion by ECs through TLR4, both of which leaded to neovascularization in tumor microenvironment. Moreover, extracellular peroxiredoxin 1 (Prx 1) has also been defined as a TLR4 ligand [72]. Riddell *et al.* [73] found that the number of vessels and overall vascular areas are increased in prostate cancer (CaP) cell line (PC-3M) shPrx1 tumors, which expressed Prx1 specific shRNA that can lead to a 50% reduction in Prx1 expression, rather than in control group in which tumors expressing non-specific shRNA. Furthermore, analysis of angiogenetic protein expression in PC-3M tumor lysates showed that VEGF secretion is notably reduced in shPrx1 cells as well as scramble cells, which both were transfected with vectors encoding a MyD88 domain negative(DN) cDNA, suggesting that TLR4-MyD88 pathway is involved in the production of VEGF mediated by Prx1 [73]. The role of TLR4 in the Prx1 induction of VEGF expression in prostate tumors has been further turned out to be dependent on the interaction of hypoxia inducible factor-1 (HIF-1) and VEGF enhancer NF-κB in tumor microenvironment [74].

Recently, in a study of the role of HMGB1-TLR4 signaling pathway in promoting EPCs recruitment in a mouse model following alkali, an increased level of EPCs recruitment was detected in injured corneas when recombinant HMGB1 and an exogenous TLR4 agonist—LPS were administered to the corneas after alkali-injured [23]. Meanwhile, this phenotype was reversed by inhibiting the activity of HMGB1 and LPS [23]. Moreover, the role of TLR4 in facilitating EPCs collection was related to over expression of a vital cytokine in EPCs mobilization, which was considered as stromal cell-derived factor 1 (SDF-1) [23]. Another study showed that biglycin (BGN), an important constituent of the extracellular matrix in variety tumors, promotes tube formation, migration and proliferation of ECs in a TLR2/4 signaling pathway [75]. Therefore, TLR4 takes an important part in facilitating the formation of vessels in tumor microenvironment through multiple mechanisms.

### MSCs expedite tumor development partially through TLR4 signaling pathway

Mesenchymal stem cells (MSCs) are multifunctional cells derived from adipose tissue, bone marrow, and some other tissues. As a type of tumor stromal cell, MSCs promote tumor growth and metastasis by secreting cytokines and chemokines in tumor microenvironment [76, 77]. Shi *et al.* [78] have first found that bone marrow-derived MSCs expressed TLR4 mRNA and functional TLR4. It has been ulteriorly demonstrated that TLR4 expression on MSCs have a positive effect on the Tcell response [79, 80] and B lymphocyte-related immune regulation [81]. Down-regulation of TLR4 signal pathways in bone marrow mesenchymal stem cells (BMSCs) gave rise to the reduction of IL-1β and TNF-α in bone tissue, thus contributed to efficient control of spinal cord inflammation [82]. Recently, Lu’s study manifested that the levels of TLR4 mRNA expression in MSCs isolated from bone marrow of patients with acute myeloid leukemia (AML-MSC) as well as from the patients’ lung cancer tissues (LC-MSC), were much higher than that in MSCs obtained from the bone marrow of healthy volunteers (BM-MSC) [83]. The secretion of IL-6 and IL-8 by the sorted TLR4-positive MSCs (TLR4+ MSCs) was lower than that by unsorted MSCs. Meanwhile, the levels of IL-6 and IL-8 was significantly upregulated by LPS and the amplified effect was larger in TLR4+ MSCs than that in unsorted MSCs [83] . However, the level of MSC-derived monocyte chemotactic protein-1 (MCP-1), was much higher in the supernatant of TLR4+MSCs, suggesting that TLR4 may play a vital role in induction of MCP-1, which has been defined as a promoter in breast cancer cell migration [84]. The expression NKG2D receptor on NK cells as well as the multiplication capacity and cytotoxicity of NK cells were both inhibited after co-culturing with TLR4+MSCs [83]. Interestingly, the suppression of NK cell cytotoxicity was more remarkable following treatment with LPS and recovered by damping the TLR4 function [83], suggesting that TLR4 expression on MSC play a crucial role in inhibiting NK cell function in tumor microenvironment [83].

### TLR4 expressed on tumor cells

It was verified that TLR4 also expresses in many type of tumors, such as hepatocarcinoma, glioblastoma, lung cancer and breast tumor [85–88]. TLR4 were found to be recognized by extracellular HSP70 in the tumor microenvironment, and it plays a positive role in proliferation and migration of tumor cells [89]. Recently, HSP70 induced an increased expression of nuclear NF-κB and an enhanced proliferation of H22 hepatocarcinoma cells, however, this function was later inhibited by resveratrol, a TLR4 signaling inhibitor [86]. In another study, EGFR transactivation, which may contribute to cell migration, induced by HSP90a in U87 glioblastoma cells was inhibited with the down-regulation of TLR4, indicating that HSP90a promotes glioblastoma cells migration through the interacting with TLR4 [85]. Furthermore, over expression of TLR4 on hepatocellular carcinoma cells was positively correlated with the number of CD4+CD25^high^FOXP3+ T_reg_ in tumor tissue, which leads to immune tolerance and tumor progression. Metadherin (MTDH) was definitely known as a protein associated with tumor progression and metastasis in cervical cancers [90, 91]. The expression of MTDH are presented at a higher levels in TLR4 positive breast cancer cells (MDA-MB-231,MCF-7,and MDA-MB-468) than that in TLR4 negative T47D cell lines after treated with LPS, suggesting the pro-tumor role of TLR4 expressed on tumor cells [92]. Furthermore, TLR4 has also been reported correlation with metastasis of breast cancer cells [93, 94] and has down favor on poor survival of breast cancer patients [95].

In addition to endogenous ligands secreted by necrotic tumor cells, TLR4 is also directly activated by paclitaxel, a chemotherapeutic drug against various human cancers. Paclitaxel brought out robust inflammatory responses and tumor growth in TLR4 positive cells, but not in TLR4 negative cells [10], indicating that TLR4 may be a underlying reason for the resistance to chemotherapy. Mallick et al. [96] have found that paclitaxel altered the expression of key enzymes involved in irinotecan metabolism in TLR4-dependent mechanism, which demonstrated that TLR4 could be a novel mediator of paclitaxel and irinotecan drug-drug interaction. Furthermore, dysregulation of gene TLR4 was associated with cisplatin resistance in human squamous cell carcinoma [97]. As previously shown, Prx1 interaction with TLR4 on CaP cells promoted prostate cancer growth through chronic activation of tumor angiogenesis [73, 74]. Consistently, knockdown of TLR4 via TLR4 siRNA in human breast cancer cell line MDA-MB-231 induced a decreased secretion of cytokines including IL-6 and IL-8, and a inhibition in proliferation and survival of these cells [98]. Meanwhile, TLR4 RNA expression in breast cancer with node metastasis is much higher than that without node metastasis [88]. These results suggested that of TLR4 on tumor cells may be a potential therapy for breast cancer.

Studies have also shown that the expression of TLR4 are higher in non-small cell lung cancers (NSCLC) than that in normal tissues and the expression of its serum soluble pattern may act as a biomarker to predict radiation pneumonia risk in local advanced NSCLC patients [99, 100]. In addition, TLR4 expression is associated with tumor stage. One study of 37 patients with stage II and III colon cancer has shown that TLR4 expression was significantly higher in stage III tumors than stage II [101]. Previous study has also found a rising tendency on the NSCLC stage from I to III [102]. Furthermore, high expression of TLR4 in human hepatocellular carcinoma (HCC) tissues has also been found associated with poor survivals in patients [103]. On the contrary, our previous study has shown that low levels of serum soluble TLR4 were associated with poor survival of early stage NSCLC patients [104]. Meanwhile, serum TLR4 levels of patients with distant metastasis was much lower than that of patients without metastasis [104]. These results suggested that serum TLR4 maybe a biomarker of tumor metastasis and prognosis. Serum TLR4 is the soluble form of its extracellular domain on cells, there is a hypothesis that the extracellular domain of TLR4 may crack from tumor cells with the development and metastasis of tumor.

## CONCLUSIONS AND PROSPECT

Although TLR4 activation on tumor cells, stroma cells as well as vascular epithelial cells definitely benefits the tumor, stimulation of this pathway in immune cells may have two reversal consequences. TLR4 expressed on DCs play a role in promoting anti-tumor immune response, but that expressed by MDSCs, vascular epithelial cells, macrophages MSCs and tumor cells play the opposite role. Furthermore, activation of TLR4 on T cells have anticancer and pro-tumor consequence in tumor microenvironment. Meanwhile, studies found that serum TLR4 maybe a biomarker of tumor metastasis and prognosis. Immunotherapy is the fourth and promise therapeutic method following surgical, chemotherapy and radiotherapy. The *in vitro* and *in vivo* experiments indicated that multiple substances functioned as immune adjuvant through binding to TLR4 on DCs, and played a promising role in anti-tumor therapy. Immunomax, a new defined TLR4 agonist, prolongs the survival of mice following primary 4T1 tumor resection by activating DCs and NK cells co-operation in tumor microenvironment [94]. In view of the “double-edged sword” role of TLR4 activation, from my own perspective, the potential role of TLR4 in tumor microenvironment needs to be further clarified.
